# The right face at the wrong place: How motor intentions can override outcome monitoring

**DOI:** 10.1016/j.isci.2023.108649

**Published:** 2023-12-07

**Authors:** Gabriel Vogel, Lars Hall, James Moore, Petter Johansson

**Affiliations:** 1 Department of Cognitive Science, Lund University, Sweden, Helgonavägen 3, 222 22 Lund, Sweden; 2 Department of Psychology, Goldsmiths University of London, Lewisham Way, New Cross, London SE14 6NW, UK

**Keywords:** Biological sciences, Neuroscience, Cognitive neuroscience

## Abstract

The concept of intentions is often taken for granted in the cognitive and neural sciences, and comparing outcomes with internal goals is seen as critical for our sense of agency. We created an experiment where participants decided which face they preferred, and we either created outcome errors by covertly switching the position of the chosen face or induced motor errors by deviating the mouse cursor, or we did both at the same time. In the final case, participants experienced a motor error, but the outcome ended up correct. The result showed that when they received the right face, but at the wrong place, participants rejected the outcome they actually wanted in a majority of the trials. Thus, contrary to common belief, higher-order outcomes do not always regulate our actions. Instead, motor “wrongness” might sometimes override goal “rightness” and lead us to reject the outcome we actually want.

## Highlights

• People rejected outcomes they wanted when they reached it through an incorrect action
• People rejected the outcome they wanted with very high confidence
• Illusory outcome rejection changed people’s later preferences
• Motor mismatches can create an illusory sense of agency for outcomes

## Graphical abstract

**Figure:**
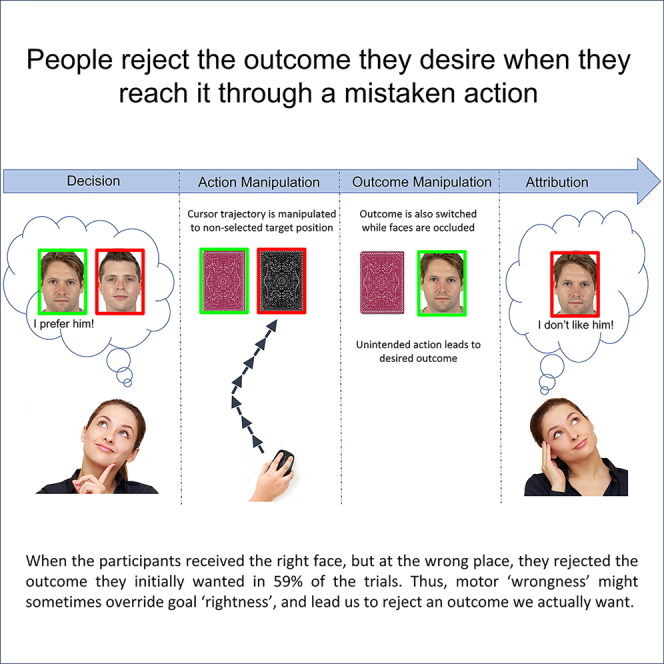

## Introduction

The everyday concept of intentions is often taken for granted in the cognitive and neural sciences.^1^^,^^2^ Consequently, a prominent class of models suppose that the brain has a clear representation of goals and that a comparison between intentions and outcomes is an essential feature of cognition. For example, many models of cognitive control are premised on the idea that “comparing outcomes with internal goals is critical for optimizing performance” and that this activity is routinely performed by the brain.^3^ These models also suggest that our sense of responsibility and agency (i.e., the sense of control of our bodily movements and their outcomes) derive from the same matching process between intended and observed outcomes.^4^^,^^5^

We have previously introduced the phenomenon of choice blindness (CB) as a challenge for these types of “comparator” models.^6^ In a typical CB experiment participants choose between different options; then a discrepancy is introduced between the intention and the outcome, and it is measured to what extent this is noticed or accepted by the participants. For example, in the original study by Johansson et al.,^6^ participants decided which of two faces they found most attractive, but a sleight of hand made it so that they were presented with the rejected option as their actual choice. The surprising finding was that participants often failed to notice this mismatch and instead proceeded to confabulate explanations for why they preferred the manipulated option they ended up with (see Somerville et al.^7^ for a review of CB research).

On the face of it, CB does not seem to fit with comparator models but instead suggests that knowledge of our own intentions might rely on unconscious inferences based on various contextual and behavioral cues, much in the way that we interpret the behavior of others.^8^^,^^9^^,^^10^^,^^11^^,^^12^ However, the interaction of motor and purported “higher-order” levels of monitoring is a severely understudied topic, and very few attempts have been made to disentangle the potential contributions from different types and levels of monitoring that might occur when people make decisions.^13^

Thus, to investigate the potential relation between motor execution and higher-order intentions, we created a novel cognitive monitoring experiment where different levels in this purported action/goal hierarchy are explicitly pitted against each other. In CB studies, the manual actions performed by the participants are typically correct (pointing, writing, clicking a mouse, etc.), but the outcome is nevertheless mismatched. This makes it difficult for participants to rely on potential error signals arising from proprioceptive and kinesthetic feedback to guide their monitoring. But what would happen if participants instead were faced with a situation where there is an error at the motor level but the outcome somehow ends up correct anyway?

According to comparator-based cognitive models, what should matter in this situation is the outcome of the choice. As Haggard (2012) puts it, “If the comparison between predicted and actual feedback generates no error, then “I did that”.”^5^ From this perspective, it should not matter whether some trouble happens during the selection process. If the outcome matches the intention, we should embrace it. However, CB has already shown that people might accept choices they did not make. Then the reverse might also be possible, where people instead *reject choices they have actually mad*e. Thus, our prediction is that prior intentions may sometimes be too imprecisely represented, left unmatched with actual outcome, or overpowered by the motor feedback. Hence, the cue of motor wrongness given by the erroneous action can take precedence when people infer their intent, leading them to wrongly conclude that the outcome is not what they wanted.

**Figure 1:**
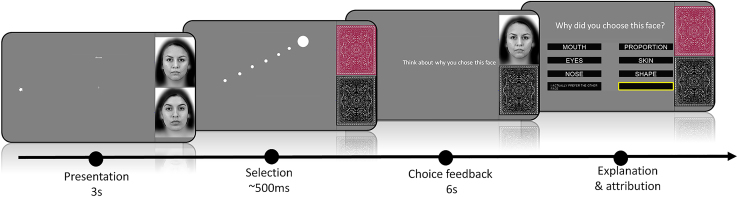
Timeline of a regular choice trial Two faces are presented for 3 s; then the participants use their mouse cursor to indicate their choice. After 6 s of deliberation, they are given six alternatives to choose from as the main driver of their choice. In addition, there is a regret button worded “I actually prefer the other face.” The traces of the previous positions of the cursor are only shown for illustration purposes. Only the current position of the cursor was visible for the participant.

To test this, we invited 80 participants to take part in a computerized decision experiment. For 38 pairs of faces, they had to choose the one they found the most attractive and explain their decision. The faces were shown briefly on the screen before they were occluded by an image of a backside of a playing card. At that moment, people reported their decision by dragging a mouse cursor across the screen to the position of their preferred face. After their selection, the purportedly selected face re-appeared and participants were prompted to reflect on why they chose it. Then, the face was hidden again, and subjects were asked to pick, among different options describing facial features, which one mattered the most for their decision. If and only if they rejected the outcome (pressing the “I actually prefer the other face button”), an additional screen appeared asking them to evaluate how confident they were in their judgment that they preferred the other face (on a 1 to 7 Likert scale). A timeline of the full procedure of the experiment is shown in Figure 1 in the following. To measure choice consistency, the participants also had to make a second choice for a subset of the previously presented face pairs at the end of the experiment.

**Figure 2:**
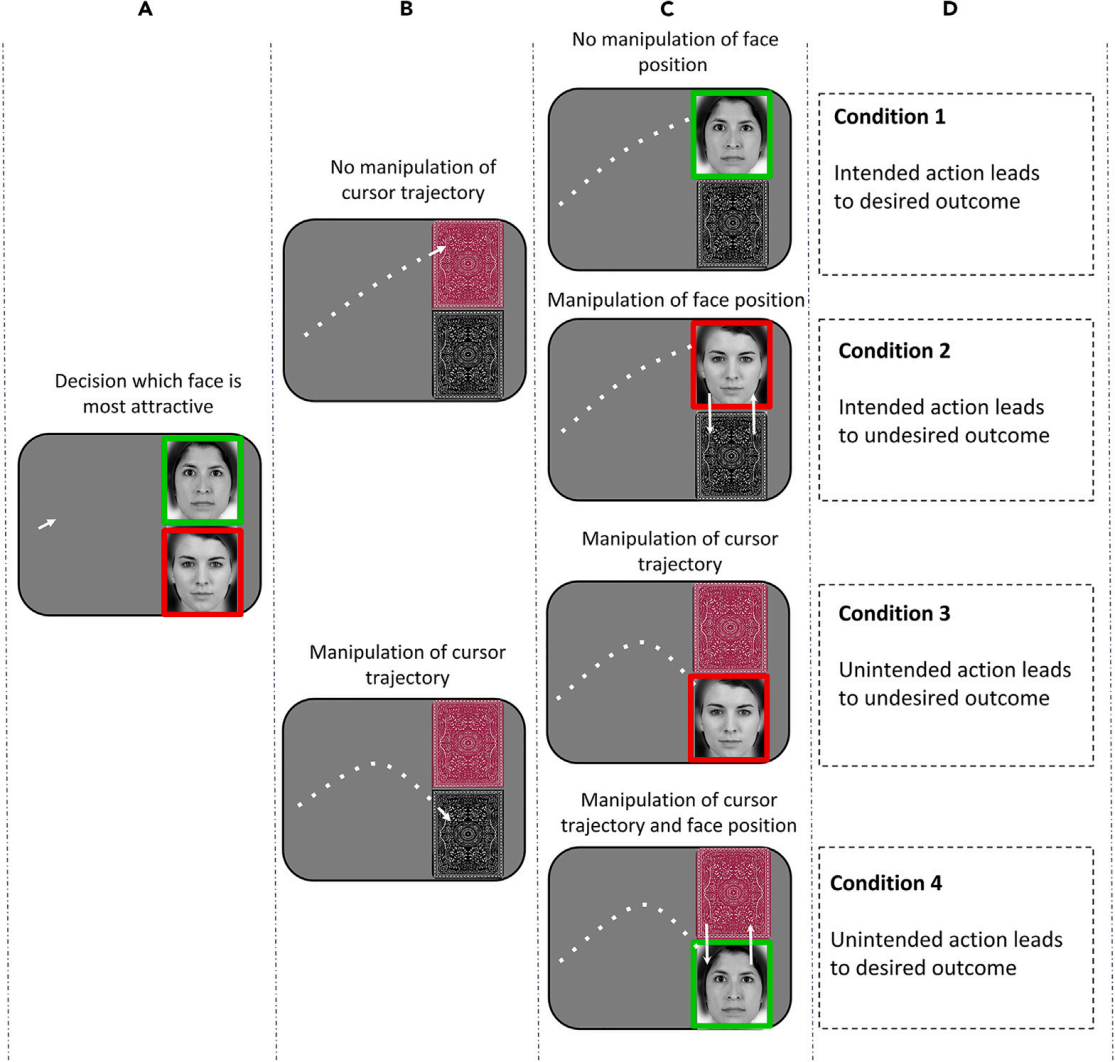
Experimental conditions (A) In each condition, participants had to choose between two faces that were displayed for 3 s. (B) Then these faces were hidden by card backs’ pictures, and people selected the face they wanted by moving a mouse cursor to the location of the desired face. (C) Once they reached it, a face was shown again at this location, as feedback. (D) The experiment had four different conditions. Condition 1 was a control condition where mouse cursor and outcome were correct. In condition 2 only the outcome was manipulated. The face that was not chosen appeared at the position of the chosen face after selection. In condition 3 the cursor was manipulated to move toward the location of the unchosen face, and the unchosen face shown as feedback. Condition 4 was the most important. The cursor was deviated toward the unchosen face, but after this wrong selection the desired face was shown as feedback. The traces of the previous positions of the cursor are only shown for illustration purposes. Only the current position of the cursor was visible for the participant.

Unbeknownst to the participants, we included three types of manipulations in the experiment. Firstly, we performed a manipulation where we switched around the faces while they were occluded. In this case the intended actions of the participants lead to undesired outcomes, and we measured how participants reacted to these mismatches. An interface button reading “I actually prefer the other face” allowed them to easily indicate whether they accepted the manipulated outcome or rejected it in favor of their original intent. In another condition, we instead introduced a motor error, by programming the mouse cursor to forcefully deviate from the trajectory aimed by the participants and select the face participants did not want. Thus, in this case an unintended action led to an undesired outcome. Again, we used the response button to measure whether the participants accepted or rejected this outcome. Finally, we combined both outcome and cursor manipulation and created a novel situation where *unintended actions lead to desired outcomes*. The complete structure of the study is shown in Figure 2 in the following.

## Results

**Figure 3:**
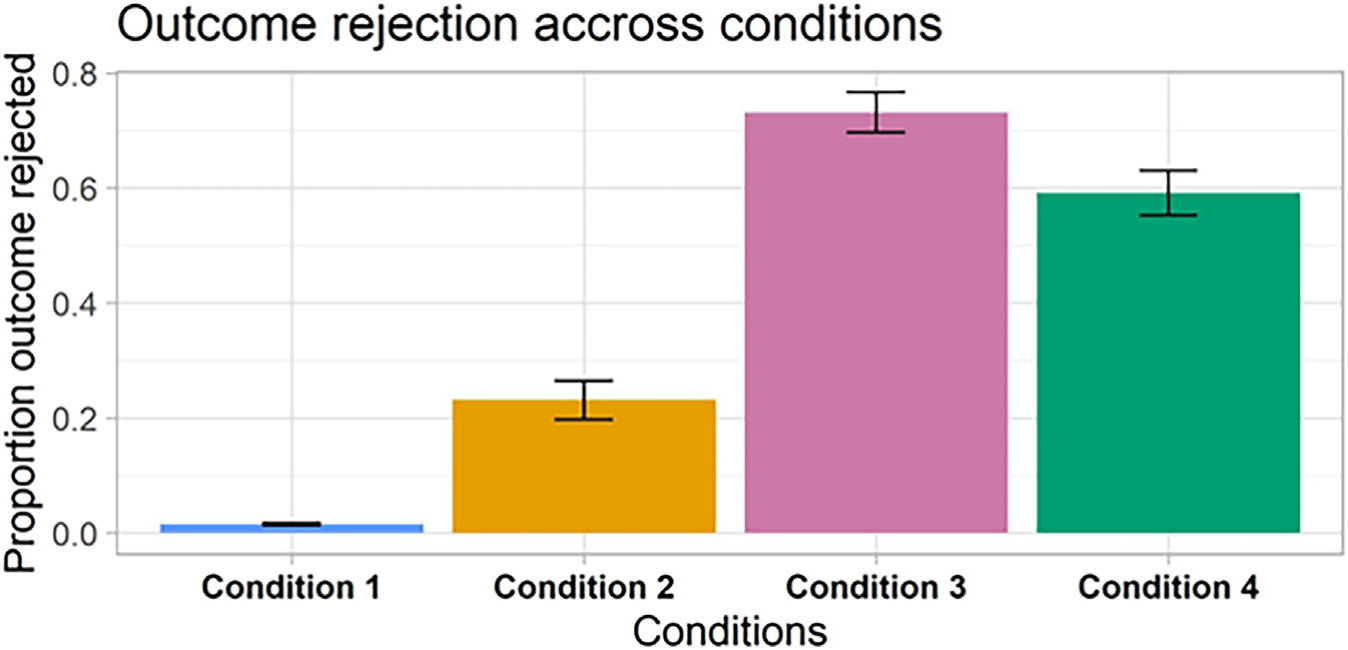
Outcome rejection rate in the different experimental conditions In condition 4, where the right face appears at the wrong place after manipulation of the cursor trajectory, participants mostly reject the outcome they actually wanted. In condition 2, where participants receive the wrong outcome without cursor manipulation, they reject it less often than they reject their own preference in condition 4. Error bars represent standard error.

Unsurprisingly, in the control condition with no manipulation, participants almost never used the button indicating that they actually preferred the other face (1.6%). In condition 2, in line with our previous research, when an intended action led to an undesired outcome, participants accepted this in a full 77% of the manipulated trials. In contrast, in condition 3, when an undesired outcome instead was achieved by way of a forced motor error, participants only accepted this in 27% of the trials. Finally, the result from the critical double manipulation in condition 4 showed that when unintended actions lead to desired outcomes, participants rejected the outcome they actually wanted in 59% of the trials (ps < 0.02, Bayes factors [BFs]>10 for all contrasts of conditions). Thus, contrary to the predictions of the comparator perspective, in a majority of the trials, the participants rejected the selected outcome if the action achieving it felt wrong (Figure 3).

**Figure 4:**
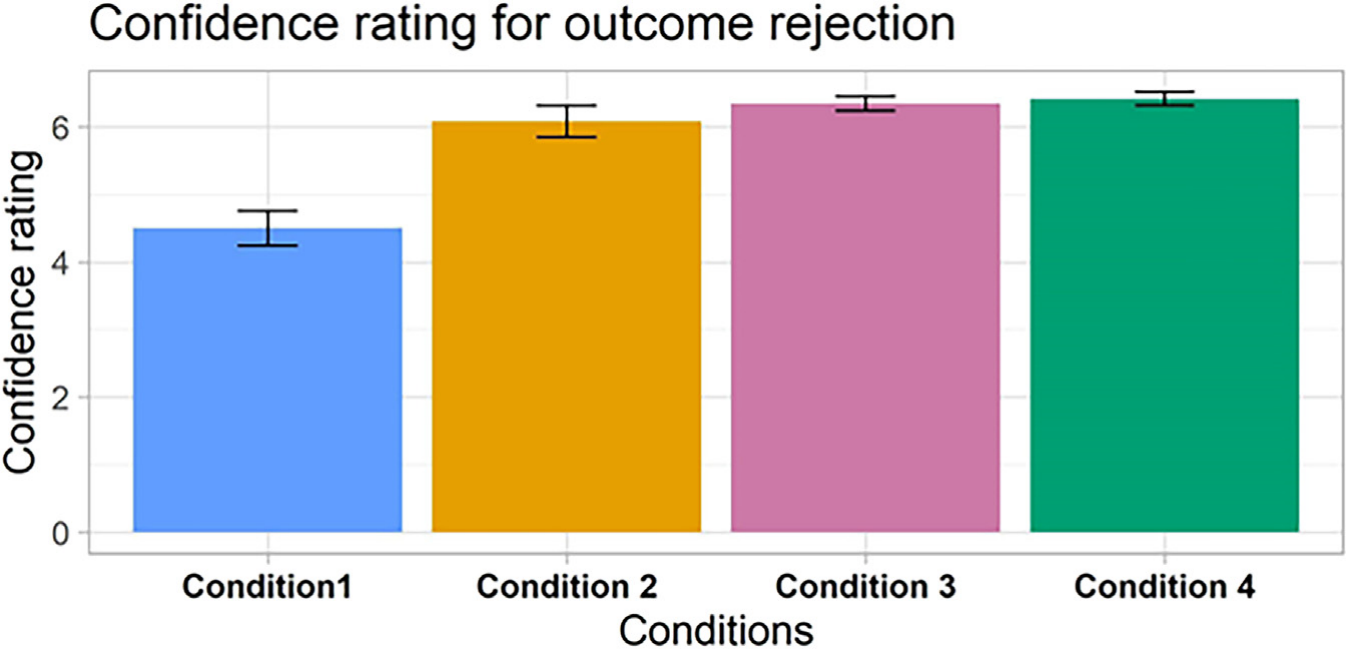
Confidence ratings for outcome rejection After rejecting the outcome, participants were prompted to rate how confident they were in their rejection (1–7 scale). They were very confident when they illusorily rejected their original preference in condition 4. This confidence was not statistically different from the confidence for outcome rejection in the other manipulated conditions (conditions 2–3). Error bars represent standard error.

**Figure 5:**
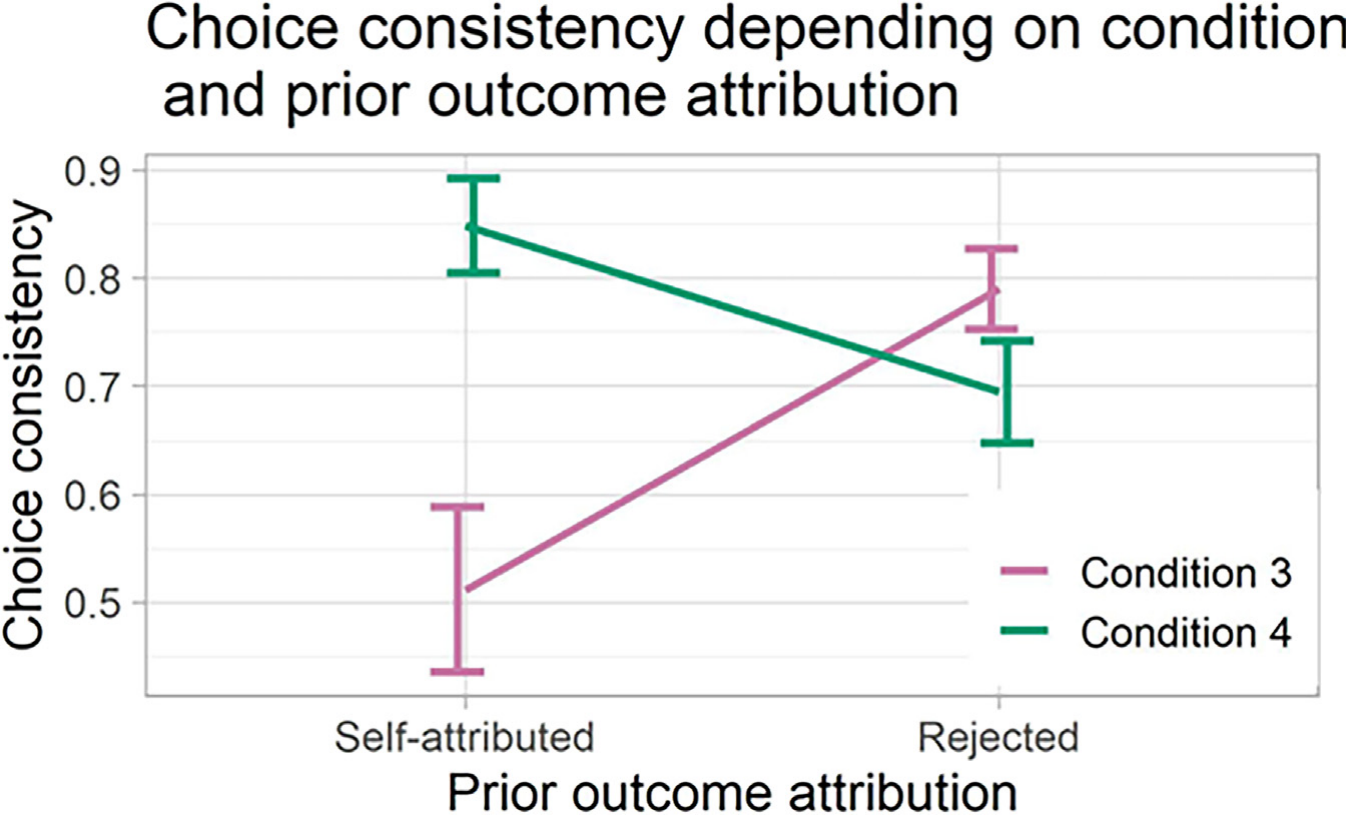
Choice consistency depending on condition and prior outcome attribution; i.e., if the participants accepted the received feedback as their own choice or not In condition 4, when participants reject the outcome they actually preferred, they become less likely to choose according to their original preference when presented with the same choice again. In condition 3, when people reject the outcome they did not want, they become more likely to choose again according to their original preference. Error bars represent standard error.

When we probed the metacognition of the participants, we found that, both in the typical CB situation and in the newly discovered *preference rejection* trials, participants were very confident in accepting the originally undesired outcomes (mean confidence rating: 6.41/7, not significantly different from the control condition, ps > 0.7, BFs<0.22 for all contrast), thus exhibiting a strong sense of agency for the manipulations (see Figure 4). Furthermore, the manipulated choices had a downstream effect on the preferences of the participants. When we asked them to choose among the same set of faces again, they were 2.59 times less likely to choose the face they originally preferred but wrongly thought they did not choose (Condition 4: confidence interval [CI] = 1.11–6.05, p = 0.027, BF = 4.16). The same pattern appeared in the other conditions, although it did not reach statistical significance in Condition 2 (Condition 3: odds ratio [OR] = 3.57, CI = 1.59–7.99, p = 0.002, BF = 11.99; Condition 2: OR = 1.15, CI = −2.04 – 2.69, p = 0.753, BF = 0.415; see SOM for more details). This is consistent with previous results that erroneous self-attributions might change people’s attitude in line with the manipulated outcomes^14^^,^^15^ (Figure 5).

## Discussion

Here we present a novel finding where participants reject desired outcomes produced by unintentional actions. In the domain of motor control, much evidence suggests that we adapt our movement in real time based on internal models, which predict the likely consequences of motor commands and match them to observed consequences (i.e., a comparator framework^14^^,^^15^^,^^16^). However, problems arise when these models are uncritically extrapolated as a mechanism for “higher-order” non-motoric outcome monitoring, and more broadly as the foundation of our sense of self and agency.^4^^,^^5^^,^^17^^,^^18^

In the context of agency processing, the interplay between lower-level and higher-level monitoring has been severely under-investigated. In one of the few studies to look at the issue, Kumar and Srinivasan (2014) adopted the so-called "event control approach," in which agent control can be realized at different levels within a hierarchical control system.^13^ In their study they showed that disruptions of lower-level sensorimotor control only reduced sense of agency when higher-level control was not achieved, i.e., when outcomes fail to match goals. Or take the model of Logan and Crump.^19^ They studied skilled typists in a serial single-word copy task, covertly corrected errors that the typists made, and inserted errors in correct responses. What they found was that the typists took credit for the corrected errors and accepted blame for the inserted ones but that their typing rate was responsive only to the actual keystrokes and thus slowed down after corrected but not inserted errors. From this they concluded that people are equipped with a hierarchical monitoring system, where an inner (motor) loop takes care of the details of performance, while an outer loop ensures that goals and intentions are fulfilled.

These findings are consistent with previous studies which also show that people tend to be unaware of sensorimotor perturbations so long as one’s goal is achieved. For example, Fourneret and Jeannerod (1998) asked people to move a stylus from a starting position to a target position.^20^ The hand was hidden from view and instead participants were shown feedback of the movement on a screen. This feedback was manipulated to varying degrees such that participants had to adjust their own movement so that it appeared to still be on course. Despite needing to change their behavior, participants seemed to be unaware of this, so long as the goal was being achieved.

In contrast to these previous studies, we find that motor errors can override goal intentions and lead us to reject the outcome we really desired, i.e., that goal achievement is not sufficient and that perturbations at the motor level do feed into higher-level aspects of action awareness. This difference could possibly be explained by the fact that in prior research^13^^,^^19^ all the crucial intentions are specified beforehand. Both the *standard* for monitoring and the *need* for monitoring are obvious for the participants (i.e., "copy the word on the screen with fidelity"). So all it really shows is that people are capable of monitoring when conditions are favorable. If we want to know something about the balance of monitoring, and how the purported "outer loop" actually works in everyday life, we must construct a setup where these factors are not built into the specifications of the task, such as in the current experiment. Thus, our results suggest that cognitive views of outcome monitoring have been relying too heavily on a postulated analogy between motor and higher-order goal monitoring.

Furthermore, our finding also has implications for theories of agency processing. A common feature of these theories is that attributions of self-agency are driven largely by correspondence between intended and actual outcomes (e.g., Blakemore et al. and Wegner^21^^,^^22^). Our finding contradicts this, with people rejecting outcomes despite a match. This suggests that a more nuanced view of agency processing is required. More specifically, it would seem that agency processing is informed not only by the comparison of intended and actual outcomes but also by other agency-related signals. One approach which possibly could accommodate our results is optimal, Bayesian precision-weighted cue integration,^9^^,^^23^ in the sense that it suggests signals coming from the motor system are more precise than those coming from outcome-monitoring mechanisms; hence, the motor mismatches dominate the precision-based inference. However, assumptions of optimality have been strongly criticized in other domains of decision making (optimal solutions can be computationally intractable, or too resource-demanding to be performed routinely^24^^,^^25^). Perhaps then, mechanisms for non-motor monitoring might operate quite differently, not relying on continuous, precise sensory predictions, or no sensory predictions at all. Indeed, higher-order goals relate to more abstract states of affairs which can have many different “sensory” realizations.^26^ Outcome monitoring could rely on so-called TOTE units (test-operate-test-exit), which only check whether some abstract environmental conditions satisfy the system’s goal.^27^ In addition, this condition check does not have to be continuous, contrary to motor control. It may be scheduled or even sometimes not even performed to save resources (resulting in well-known lapses of action, such as omissions^26^). Thus, it is possible that proper outcome monitoring requires deliberate efforts and strategies and that it is not performed automatically. Instead, the system may take what happens at the motor level as a proxy for goal satisfaction. For example, the influential dual-system framework suggests that our brain often relies on fast, cheap, but error-prone, heuristics.^24^^,^^28^ Our results might be consistent with this interpretation if participants could be shown to rely on a heuristic like “if my cursor went to the wrong position, this cannot be my choice.” Additional analyses of our data give at least plausibility to this hypothesis, although being inconclusive (see SOM). Heuristic judgments are often characterized by being faster and having lower accuracy.^29^^,^^30^ In line with this observation, we saw that inaccurate attributions tended to be performed progressively faster throughout the experiment, while accurate ones tended to become slower. However, if the accuracy of judgment descriptively increased throughout the experiment, from 43.1% (SE = 3.2%) to 48.3% (SE = 3.2%), statistical evidence was inconclusive (OR = 1.14, CI = −1.03 – 1.33, p = 0.110, BF = 1.34). It is an interesting hypothesis that increased accuracy could stem from a transition to more accurate and slower reasoning processes as the experiment progresses. We cannot satisfactorily conclude this from our data, but further experiments could test this hypothesis. Going further, new experiments could also compare optimal precision-weighted models, models with suboptimal weights, or purely heuristic models, by independently controlling the detectability of cursor and outcome manipulation in order to estimate how they are integrated in inferences of intentionality. For example, would the contribution of the motor error signal be graded, depending on how precise the motor cue is, as predicted by Bayesian optimal models of agency,^9^ or would we see that, as soon as the cursor manipulation is detectable, participants fully rely on it, as a heuristic model would suggest? A more extended modeling effort would be required to arbitrate different theories of agency judgments.

One may wonder to what extent social desirability played a role in our experiment. This question has been extensively studied in CB research. It has been repeatedly shown that social desirability is not associated with CB,^31^^,^^32^^,^^33^ nor with compliance.^32^ In addition, people who fail to detect manipulations tend to exhibit a form of “choice blindness blindness.” Post-experimental interviews showed that people believe they would not fall prey to CB if they were exposed to such manipulation, despite the fact that they unknowingly did during the experiment (see Johansson et al.^6^ and SOM). In addition, CB-induced preference change suggests that illusory self-attributions or rejections of choices are integrated into people’s preferences.^34^^,^^35^^,^^36^^,^^37^^,^^38^ This preference change can even be long lasting, persisting at least one week after the experiment.^36^ Hence, it is very likely that illusory attributions and related preference change observed in our experiment reflect a genuine phenomenon.

It is interesting to ponder how this preference change effects arise in relation to (illusory) attributions, in the present experiment and in classic CB. This preference change could arise from cognitive dissonance, people trying to resolve the conflict between their false beliefs about their choice and actual preference by changing their preferences accordingly.^39^ Memory process may be at play. It has been shown that the mere illusion of choice provides a memory boost.^40^ Reinforcement learning can also contribute to self-attribution-induced preference change.^41^ The updating of item value may be misattributed to the false feedback. One can also hypothesize that the need for consistency may lead people to repeat choices they think they made, even if they did not. In line with this interpretation, it has been shown that people strive to respect abstract principles of rationality in their choices, including consistency.^42^ Interestingly, the preference for consistency did not seem to influence false-feedback detection in CB,^43^ but whether it correlates with the magnitude of CB-related preference change has not been studied yet.

The effect of choice consistency on the tendency to reject outcomes would also be valuable to investigate. Indeed, choice consistency can also reflect preference strength,^44^ which has been shown to influence detection in standard CB studies.^36^^,^^38^ However, our measure of choice consistency was not suited for such a purpose. The reason is that illusory attributions have already changed people’s preferences when we ask them to choose from the same pairs of face a second time. Further experiments assessing preference strength with ratings or repetition of the choice problems before manipulations would allow to study this aspect of preference rejection more closely.

In addition, further explorations of the relationship between confidence, metacognition, agency, and preference change would be interesting,^45^^,^^46^ especially as our result goes against evidence that metacognition generally tracks performance.^47^ Despite showing overconfidence in illusory preference rejection, we could not investigate this effect further in our experiment. We only probed participant confidence when they rejected an outcome but not when they self-attributed it. In doing so, we tried to avoid making participants too suspicious about the real purpose of the task and the existence of manipulations. However, new methodological advances have shown that people can still remain choice blind when explicitly instructed to detect manipulations.^38^ This could be a promising avenue to connect our paradigm to previous work by Metcalfe and Greene (2007), where participants played a simple cursor video game and sense of agency was probed by explicitly judging the amount of control felt in relation to different task variables (such as speed of scrolling, density of targets, feedback of performance, etc.).^48^

On a more general level, we normally think about intentions as the goal that caused someone’s action, the goal being a “states of affairs that an agent aims to achieve.”^49^ This is also evident in the way our judicial systems define intent as “the production of a result with the aim to bring it about.”^50^ This concept derives from our folk psychological theories about how agents function based on beliefs and desires.^51^ Current evidence suggests that this folk psychology (also called theory of mind or mentalizing) is rooted in innate cognitive modules, as evidenced by the highly stable and orderly stages at which children progressively gain mastery of concepts such as desires and beliefs.^52^^,^^53^ However, even if folk psychology is crucial instrument for our daily interactions, this does not necessarily mean it will map on neatly to neurocognitive findings and provides an accurate scientific depiction of how the brain produces behavior.^1^^,^^51^^,^^54^^,^^55^ The current finding that people can come to reject what they desire if they acquire it in an unintentional way serves as a reminder that little is known about “higher-order” intentions in everyday life and calls for a renewed effort to uncover what intentions really are in the brain.

### Limitations of the study

A limitation worth mentioning is that we did not experimentally test how the preference rejection effect we report generalizes to other kind of judgments. Maybe some decisions could rely on more rational processes or bear more self-relevance, which could reduce the preference rejection effect. However, given the evidence that CB was replicated in many contexts, such as financial, consumer, political, and moral decisions,^34^^,^^36^^,^^56^^,^^57^ we believe that it is a reasonable assumption that the preference rejection effect would generalize in a similar way. Empirically investigating this question would be very valuable, especially in the context of the sense of agency, in which value-based decisions have been largely neglected. Our present paradigm is a promising tool to explore such an avenue.

## STAR★Methods

### Key resources table

**Table:** 

REAGENT or RESOURCE	SOURCE	IDENTIFIER
**Deposited data**		

Analyzed data	This paper	
Raw data and analysis scripts	https://osf.io/fr7uv/?view_only=d487f3a4e0ab43ab988c4b18c3cf4a15	

**Software and algorithms**		

Code of the experiment	https://osf.io/fr7uv/?view_only=d487f3a4e0ab43ab988c4b18c3cf4a15	
Psychopy	https://www.psychopy.org/	
R	https://www.r-project.org/	
Python	https://www.python.org/	

### Resource availability

#### Lead contact

Further information and requests for resources should be directed to the lead contact, Gabriel Vogel, Department of Philosophy and Cognitive Science, Lund University, Helgonavägen 3, 222 22 Lund, Sweden. Email: gabriel.vogel@lucs.lu.se.

#### Materials availability

Material: The material included in the study is publicly available at https://osf.io/fr7uv/.

#### Data and code availability

• Data: The dataset generated during this study is available at https://osf.io/fr7uv/.
• Code: The statistical codes used for data analyses are available at https://osf.io/fr7uv/.
• Any additional information required to reproduce the study and reanalyze the data reported in this paper is available from the lead contact upon request.

### Experimental model and study participant details

We recruited a total of 80 participants (48 females) with an average age of 26.1 years (SD= 9.8). The experiment took place in a quiet room in the Humanities lab at Lund University. Participants received one cinema voucher in exchange for their participation. At the start of the experiment, we described the general purpose and the outline of the experiment, but without telling the participants that some of the feedback on their choice or cursor trajectory would be manipulated. We also informed the participants that they could quit the experiment at any time and request their data to be erased. All participants were fully debriefed at the end of the experiment, before consenting to their anonymized data to be used by signing a consent form. The experiment was approved by the Lund University Ethics board, D.nr. 2016-1046. This work was not part of a clinical trial. Our study was not designed to investigate gender or sex differences; hence we did not analyse it. We did not collect data on the ancestry, race, or ethnicity of the participants.

### Method details

#### Procedure

The experiment consisted of three phases.

##### Training session

First, participants underwent a training session to learn to control the cursor as required for our cursor manipulation method to be successful. For training purposes, two images with A and B written on it, respectively, were displayed on the right side of the screen at the same place experimental stimuli were later displayed. Their position (top or bottom) was randomized. Participants were tasked to always select the letter A by dragging the mouse cursor over it. They were further instructed to make smooth and straight trajectories toward the centre of the target image with fast movement. After reaching the target, the cursor disappeared and a feedback screen was displayed for 2 seconds indicating whether the cursor speed had been correct, too fast or too slow, with a green, blue or red background, respectively. The desired selection time was between 100 and 250 ms and participants were told they would learn it thanks to feedback. Participants each completed a total of 20 such training trials.

##### Choice blindness phase

During the choice blindness phase, participants had to choose the face they found the most attractive and explain their choice by selecting a facial feature among a set of predefined options. This explanation served to register if participants rejected the outcome. In each trial, they were first shown two faces for three seconds. The faces were then hidden with images of card backs until participants made their selection. Participants selected their preferred face by dragging a mouse cursor over it, respecting the same requirement as in the training phase (fast, straight, and smooth movement toward the image’s centre). The image was selected when the cursor touched the pixels within the image. The trace of the previous positions of the cursor did not stay on the screen. 500ms after the cursor reached the image, a choice feedback screen was displayed for 6 seconds, showing the purportedly chosen face at the position reached by the cursor. Consecutively, the feedbacked face was hidden again by the card back and 8 buttons appeared as well as instructions which read: “Why did you choose this face? Move the selection rectangle with the arrows to the feature which best reflects your reasons. Press enter to select it”. Among the 8 buttons, six had facial features written on it, in reading order: “Mouth”, “Proportion”, “Eyes”, “Skin”, “Nose”, “Shape”. They served as decoys in order to prevent the participants from suspecting that the main purpose of the task was about self-attributions of choice outcomes. The seventh most important one read “I actually prefer the other face” and allowed the participants to reject the feedbacked face as not being the desired one. This wording was chosen as prior experiment showed that it was not exceedingly increasing suspicion while being very selective to attribution judgment. Indeed, participants only pressed this button 1.6 % of the times in non-manipulated trials. The last one was blank and served as the starting point of the selection rectangle. If participants pressed the “other face” button, they were shown an additional screen asking them to state how confident they were that they preferred the other face on a 1 to 7 Likert scale anchored with “not at all confident” and “completely confident”. Finally, they received feedback on their selection speed as in the training phase to ensure they would keep on complying with the constraints related to cursor movement.

To dissociate the contribution of motor and non-motor monitoring in people’s judgments, we introduced four conditions: i) In condition 1, the cursor trajectory and the face shown as feedback were correct. It served as a control condition. ii) In condition 2, the cursor trajectory was correct, but the face shown during the choice feedback was the one that the participant did not select, as in the classic choice blindness experiments. iii) in condition 3, the cursor was manipulated to select the face that the participant did not want, and the feedbacked face was consequently the unchosen one. iv) in condition 4, the cursor was also manipulated to select the position of the non-preferred face, but the face presented as feedback was the one that the participant had originally wanted. In a within subject design, each participant underwent each condition twice. The three conditions were presented in two blocks containing each of the conditions, and their order was counterbalanced within and between subject with a Latin square. The first manipulation condition always occurred at the 8^th^ trial, and the trial position of the ulterior conditions was pseudo randomized. The three subsequent trials after a manipulation were never manipulated, and the next manipulated trial was randomly set to occur in one of the three trials following the non-manipulated ones. In doing so, we attempted to reduce the build-up of suspicion about the real task’s purpose. Each participant completed a total of 38 choices trials during this phase. Given the constraints exposed above for avoiding suspicion, participants only were exposed to 2 trials per condition (except for condition 1, the non-manipulated one, which, also serving as filler between manipulated trials, included 32 trials). To take into account this low number of trials per participant, we used Bayesian statistics to quantify the strength of evidence given our sample size. Among the 11 statistical tests giving a bayes factor (BF) above 1 (evidence against the null), 54% gave extreme evidence (BF>100), 18% gave very strong evidence (100>BF>30), 18% gave strong evidence (30>BF>10), and 9% gave substantial evidence (10>BF>3). For the 4 BFs below 1 (evidence for the null), 25% gave strong evidence (0.1>BF>0.033), 50% gave substantial evidence (0.33>BF>.1), and 25% gave anecdotal evidence (1>BF>0.33). Overall, our study appeared well powered to give good evidence for most test, although it seemed better at safeguarding against false positives than false negatives. All BFs are reported alongside each statistical test in the main text and SOM.

##### Second choice

Finally, participants were asked to choose again among a subset of previously presented face pairs the face they find the most attractive. This aimed to assess whether illusory rejection of their own choice would change people’s preferences. All manipulated pairs were presented again, as well as six randomly selected non-manipulated pairs which served as a control, for a total of 12 trials. The trial position of the pairs was randomized. The trial structure was the same as in the choice blindness phase, except that the trial ended after the selection. No choice feedback was presented, and no manipulation occurred.

#### Material, stimuli

Participants were seated 1 meter away from a 60Hz HP E243i computer screen. The experiment ran on a computer (HP laptop 15-bs0xx) connected to a keyboard, mouse and computer screen. They controlled a cursor with a HP MODGUO mouse and the table was covered with a large mousepad to ensure smoothness of mouse movements. The face stimuli used were male (N=40) and female (N=36) faces from the Chicago face database.^58^ Faces were paired to be relatively similar. The trial position of face pairs and whether they would be part of a manipulated trial were randomized.

##### Cursor manipulation

The experiment was programmed with Python and Psychopy. The starting point of the mouse cursor was 17cm (on screen) away from the centre of images’ left edge on the x axis. On the y axis, its starting position was exactly between the 2 pictures. The cursor manipulation worked as follows: we first predicted the desired target (up or down). When the cursor had crossed a position on the x axis representing one third (5.7cm on screen) of the distance between starting position and targets, the system checked the cursor’s y position. If it was more than 5 pixels below the middle of the screen, it predicted that the lower target was aimed to be selected; if it was more than 5 pixels above, the upper target was predicted. If conditions were not met, the program waited for the next cursor position to make its prediction. Once the prediction was issued, a straight path between the current cursor position and the centre of the unwanted target was computed, and the cursor was constrained to appear following this path. The prediction of the desired target was very reliable, as it had 99.4% of accuracy (calculated on non-manipulated trials).

### Quantification and statistical analysis

All statistical analyses were performed with R and the packages lme4, lmerTest and emmeans. All statistical tests were based on mixed effects models with random effects for subjects and items.
