# Supplementary material for: The right face at the wrong place: How motor intentions can override outcome monitoring
Source: iScience. 2023 Dec 7;27(1):108649. doi: 10.1016/j.isci.2023.108649 (PMC10753065; doi:10.1016/j.isci.2023.108649)
Supplement: Document S1. Figures S1–S5 [file mmc1.pdf]

iScience, Volume *27*

Supplemental information

The right face at the wrong place: How motor intentions can override outcome monitoring

Gabriel Vogel, Lars Hall, James Moore, and Petter Johansson

Supplementary online material

Outcome rejection rate

As expected, participants mostly rejected their preference when they received their intended outcome through a manipulated cursor movement. Indeed, 59% (SE=3.9) of trials in the M condition resulted in an intentionality rejection. We ran a generalized linear mixed effect (glme) at the trial level with participant and face pair as random factor, intentionality rejection as dependent variable, and manipulation conditions as fixed effects. A comparison of marginal means without Tuckey adjustment on our model revealed a statistically significant difference between all the contrasts (Condition 2-Condition 4: estimate= -1.957, SE=0.3, p<0.001, BF>100 ; Condition 2-Condition 3: estimate= -2.752, SE=0.31, p<0.001, BF>100 ; Condition 4-Condition 3: estimate= -0.795, SE=0.28, p=0.019, BF=10.31)

Metacognitive judgment of confidence

When rejecting their intentionality, participants were asked how confident they were in their judgment that they preferred the other face. Participants displayed a very high illusory over confidence when rejecting the outcome they truly wanted in the Condition 4. Indeed, confidence ratings for rejection were the highest in the Condition 4 (mean=6.41, SE=0.10), very close to the maximum allowed by our discrete confidence scale (max=7). Confidence ratings were very high as well in the other conditions, where they related to accurate outcome rejections (Condition 3: mean= 6.34, SE=0.11 ; Condition 2: 6.08, SE=0.23). Analysis of marginal means from a linear mixed effect model (LME) showed no statistically significant difference between any of these contrasts (Condition 2 -condition 4: estimate=-0.208, p=0.352, BF=0.218; condition 2 – condition 3: estimate=-0.201, p=0.386, BF=0.207; condition 4 - condition 3: estimate=0.014, p=0.880, BF=0.093; all ps without adjustment).

However, this is not to mean that participants always mindlessly selected the highest confidence value available on the scale. In the few non manipulated trials (Condition 1) in which participants pressed the button we used to assess outcome rejection, their confidence was lower (mean= 4.5, se=0.26) and contrasts of marginal means in a LME including NM trials showed that the confidence ratings in NM were statistically lower than in each other conditions (all ps<0.001 with Tuckey adjustment, all BFs>100). Hence, our results reflect a genuine lack of metacognitive sensitivity of intentionality rejection to outcome match in the M condition, not a lack of scale sensitivity.

Choice consistency and preference change

To assess whether incorrect attribution of choice outcome would change people's own preferences, we asked to people to choose again the face they preferred among the manipulated pairs and the same number of randomly selected non manipulated

ones. Hence, we could estimate preference change by looking for a modulation of choice consistency (the likelihood to choose the same alternative during the second choice) depending on prior intentionality judgments. We ran a GLME model with participant and face pair as random effects, choice consistency as dependent variable, and manipulation conditions and prior outcome attribution as fixed effects. We saw a statistically significant effect of intentionality rejection on choice consistency in the Condition 3 and Condition 4. In the Condition 4, choice consistency decreased when participant rejected the outcome they actually wanted during previous choice (OR= -2.59, CI= -6.05 – -1.11, p=0.027, BF=4.16). Conversely, in Condition 3, choice consistency increased when participant reject the outcome, not being the one they originally selected (OR=3.57, CI= 1.59 – 7.99, p=0.002, BF=11.99). We ran an additional GLME with an interaction between the conditions 3 and 4 and prior attribution, and saw a significant interaction between them (OR=9.62, p<0.001, BF>100).This was to be expected as participants reject their true preference in Condition 4, hence become less likely to choose it again. Conversely, in Condition 3, they reject a manipulated outcome, hence they are more likely to repeat their prior true choice.

Surprisingly, however, no effect of prior intentionality judgment on choice consistency was observed in the Condition 2 (OR=1.15, CI= -2.04 – 2.69, p=0.753, BF=0.415). Nevertheless, there was indeed an effect of the manipulation on choice consistency in Condition 2, disregarding further modulation by attribution judgment. Choice consistency in Condition 2 was lower than in the non-manipulated Condition 1 (66%, SE=3.7 VS 78.8%, SE=1.8). A GLME showed that the difference in choice consistency between Condition 1 and 2 was statistically significant (Condition 1-Condition 2: OR= -2.01, CI=-3.01—1.34, p<0.001, BF=27.17).

Preliminary evidence for heuristic and deliberate agency judgments: analysis of reaction time

We suggested that preference rejection may rely on a heuristic of the sort: “if my cursor went to the wrong position, this cannot be my choice”. Intuitive judgments are often characterized by being faster and having lower accuracy[1,2]. To explore this possibility, we analysed how reaction times (RTs) for outcome attribution relates to the correctness of the attribution judgment. Our hypothesis was that people may rely on fast heuristic judgments when they are confronted to the first manipulations. Indeed, they don’t expect that a wrong cursor trajectory could lead to the right outcome. But, as the experiment goes, participant may start to expect manipulations, becoming more attentive and careful in their reasoning. Hence, they may take more time to inhibit their intuitive response and become more likely to attribute it correctly.

This is broadly the pattern we see. The reaction time for incorrect intentionality judgments decrease throughout the experiment, while the reaction time for correct ones increases (interaction: OR=1.11 , CI=1.05 – 1.17 , p< 0.001, BF=63.62; see figure S1). This can be interpreted as a progressive switch from intuitive to deliberate judgment as the experiment progresses.

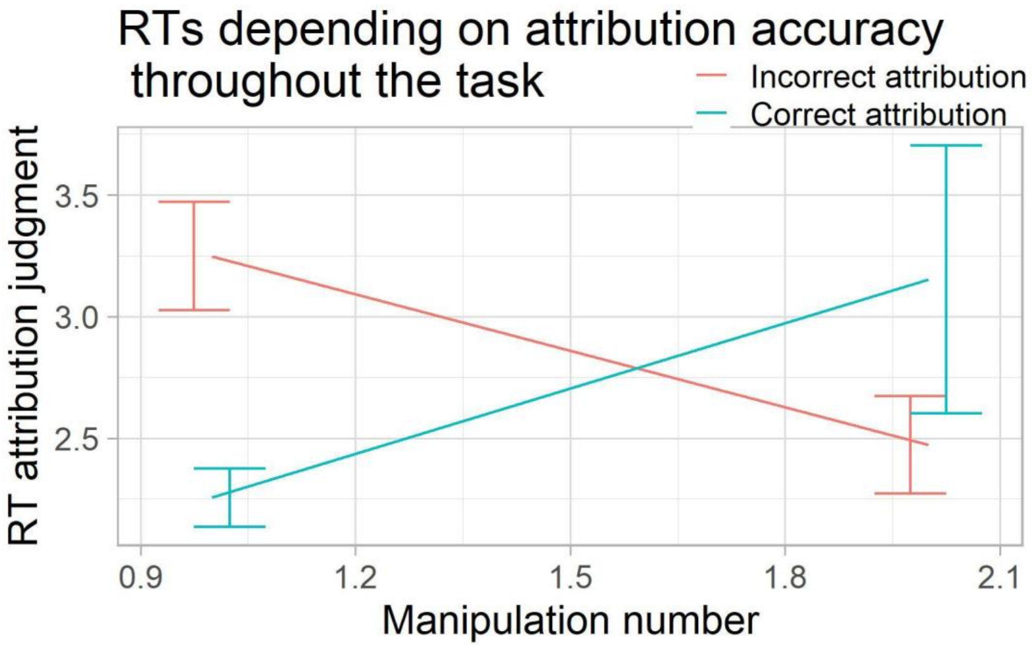

Figure S1. Reaction times (in seconds) for attribution judgments of outcome depending on whether the judgment is correct or not, related to Figure 3.

However, if the accuracy of judgment descriptively increased throughout the experiment, from 43.1% (SE=3.2%) to 48.3%(SE=3.2%), statistical evidence was inconclusive (OR=1.14, CI=-1.03 – 1.33, p=0.110, BF= 1.34; see figure S2). It is an interesting hypothesis that increased accuracy could stem from a transition to more accurate and slower reasoning processes as the experiment progresses. We cannot satisfactory conclude this from our data, but further experiments could test this hypothesis.

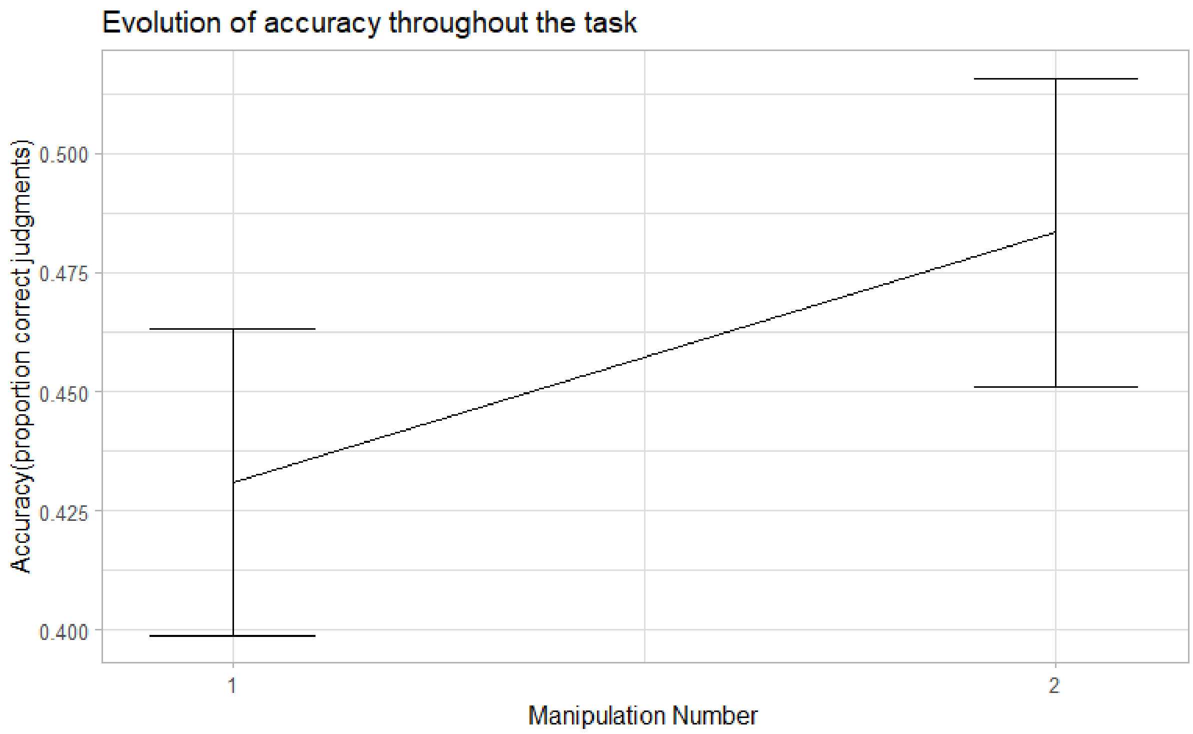

Figure S2. Evolution the accuracy of outcome attribution judgments. Participants make less attributions errors during the second block of manipulations, related to Figure 3.

Cursor trajectories

To analyse the cursor trajectories, we used the package mousetrap and custom scripts in R. We first visually inspected all the trajectories to exclude the ones that were aberrant, reflecting cursor dysfunctions. We excluded 8/320 trajectories, which only belonged to the conditions with cursor manipulations (3 and 4). We then symmetrized the trajectories to be able to aggregate trajectories going to the upper and lower targets. We also interpolated the trajectories between the different recorded positions and normalized the time of the trajectories. To see whether participants tried to correct the cursor's trajectory when we introduced a manipulation, we plotted the aggregated trajectories in each condition (see figure S3).

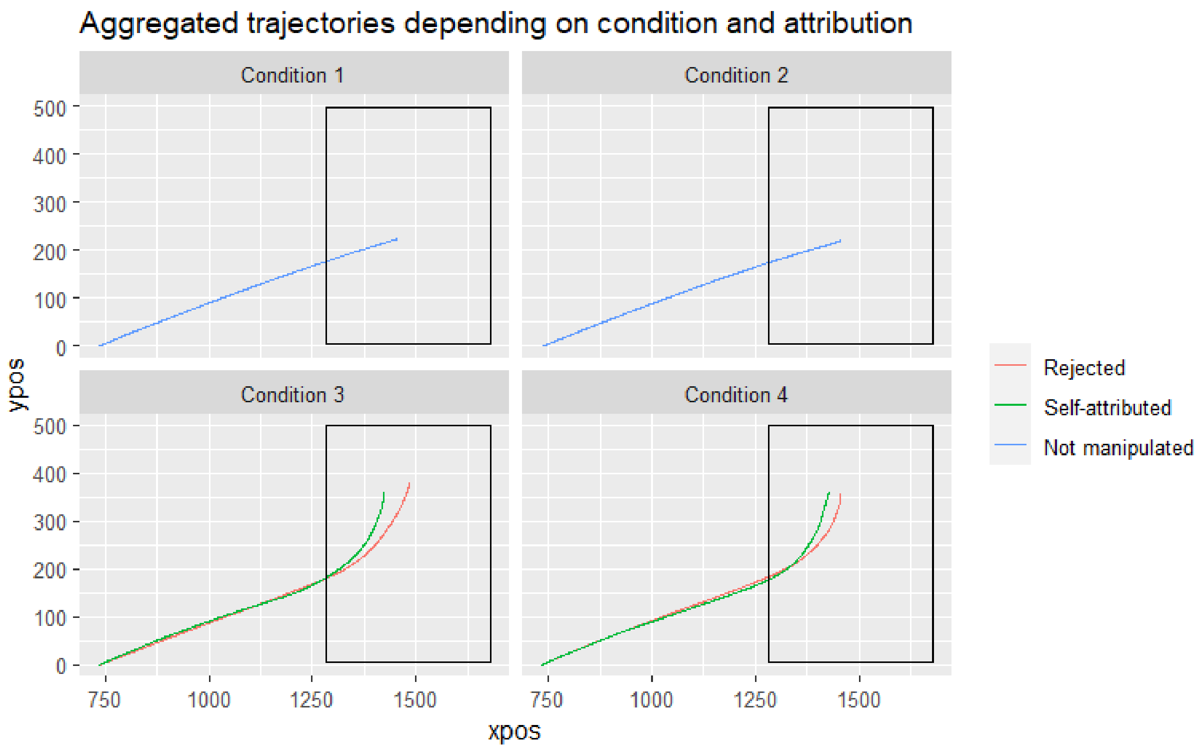

Figure S3. Aggregated real mouse cursor trajectories in each experimental condition, depending on whether the cursor was manipulated, and the outcome self-attributed, related to Figure 2.

As expected, in condition 1 and 2, where the cursor is not manipulated, participants follow a straight line without trajectory correction. However, in condition 3 and 4, when the cursor trajectory is manipulated, participants try to compensate the cursor deviation by dragging it in the opposite direction (e.g. they want to select the upper target, the cursor goes down, so they try to move the cursor even more up). So, cursor manipulations were indeed processed and acted upon at the motor level.

To assess more precisely the presence of corrective movements, we calculated the magnitude of upward movement (after symmetrisation of trajectories) once the cursor reached the target for each trajectory. We found a significant difference in corrective movement between conditions where the cursor was manipulated, versus not ($t=17.38$, $df=319.24$, $p< 0.001$; see figure S4). However, we did not find a difference between rejected and non-rejected outcomes using this method ($t=-0.45$,$df=183.46$, $p=0.66$).

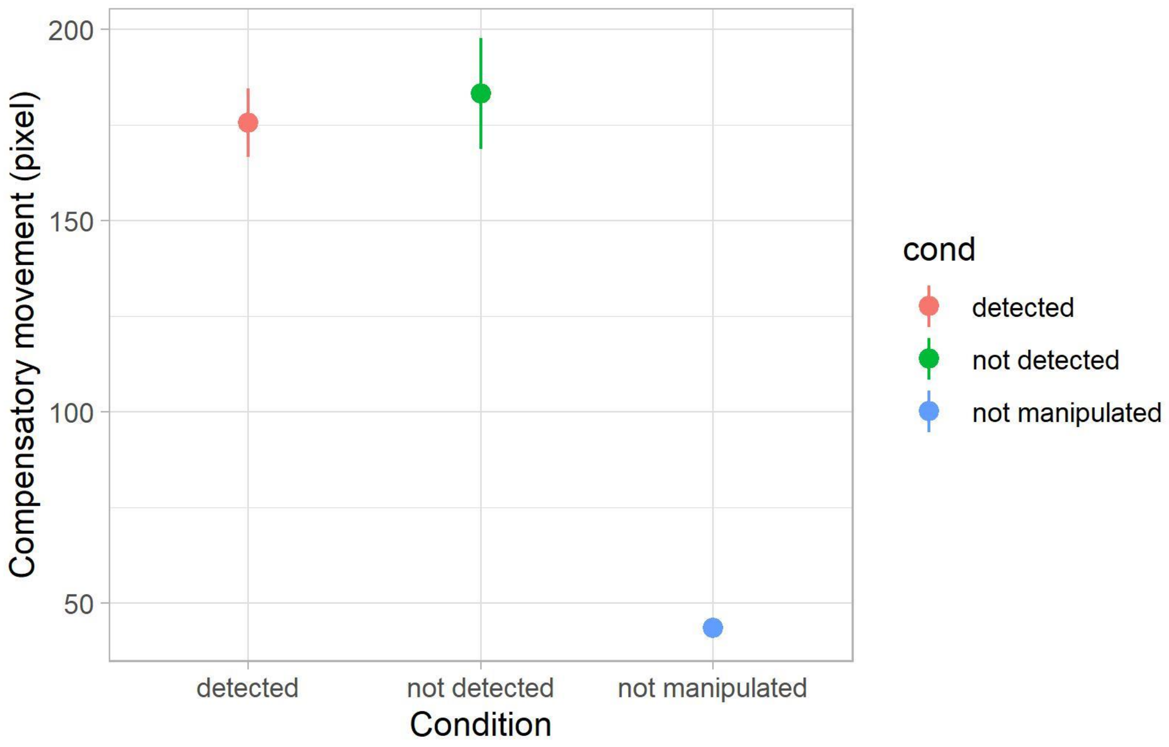

Figure S4. Amount of compensatory cursor movement depending on cursor manipulation and outcome attribution, related to Figure 2.

Interestingly, one may wonder whether the magnitude of mismatch between actual and desired cursor position predicts outcome rejection. Indeed, from a comparator perspective, the sense of agency depends on whether and how much error signals have been generated by internal models for motor control. To test this, we computed a “motor error” for each trial in conditions with cursor manipulation (3 and 4). For each recorded cursor position, we calculated the distance on the y axis between the apparent manipulated cursor position and the one where the cursor should have been if no manipulation had happened (only the y position was manipulated).

Results were inconclusive. Descriptively, the magnitude of motor error tended to be higher when participant rejected outcomes (see figure S5). However, this effect only reached statistical significance in condition 4, and Bayesian analyses suggested a lack of conclusive evidence in both conditions (condition 3: OR=1.71, CI=0.27 – 9.77, p=0.513, BF=1.06; condition 4: OR=2.1, CI=0.37 – 9.44, p=0.013, BF=1.40). This may be explained by a lack of statistical power. This can also relate to the fact that cursor trajectories were very fast (mean=273ms, SD=567ms) and stereotypical (participants were instructed to move the cursor in a straight line toward the centre of their desired target), which impeded the differentiation of different levels of motor errors.

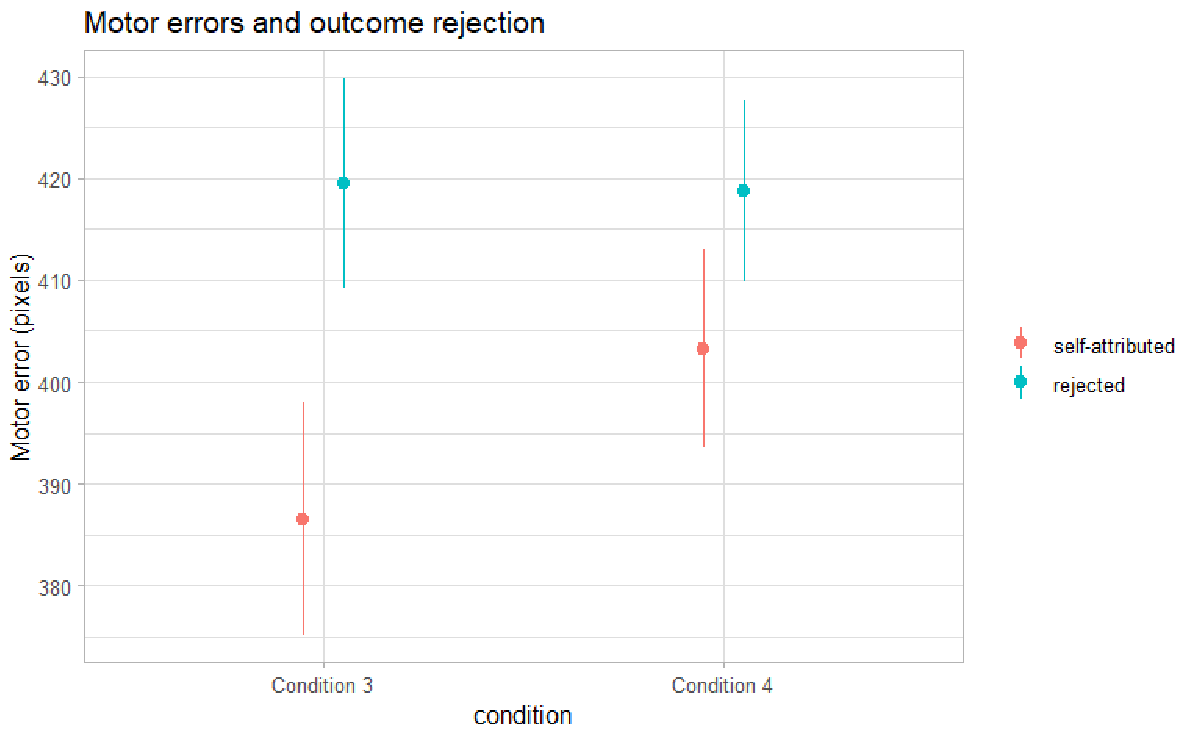

Figure S5. Relationship between motor error and outcome rejection in conditions in which the cursor is manipulated (Conditions 3 and 4), related to Figure 2.

Bibliography

1. De Neys, W. (2006). Automatic-heuristic and executive-analytic processing during reasoning: Chronometric and dual-task considerations. Q J Exp Psychol (Hove) *59*, 1070–1100. 10.1080/02724980543000123.

2. Evans, J.St.B.T., and Stanovich, K.E. (2013). Dual-Process Theories of Higher Cognition: Advancing the Debate. Perspect Psychol Sci *8*, 223–241. 10.1177/1745691612460685.
